# Artificial Intelligence Models for Predicting Stock Returns Using Fundamental, Technical, and Entropy-Based Strategies: A Semantic-Augmented Hybrid Approach

**DOI:** 10.3390/e27060550

**Published:** 2025-05-23

**Authors:** Gil Cohen, Avishay Aiche, Ron Eichel

**Affiliations:** School of Management, Western Galilee Academic College, Acre 2412101, Israel; avishaya@wgalil.ac.il (A.A.); rone@wgalil.ac.il (R.E.)

**Keywords:** artificial intelligence, trading, fuzzy logic, technical, fundamental

## Abstract

This study examines the effectiveness of combining semantic intelligence drawn from large language models (LLMs) such as ChatGPT-4o with traditional machine-learning (ML) algorithms to develop predictive portfolio strategies for NASDAQ-100 stocks over the 2020–2025 period. Three different predictive frameworks––fundamental, technical, and entropy-based––are tested through examination of novel combinations of ML- and LLM-derived semantic metrics. The empirical results reveal a considerable divergence in optimal blending methods across the methodologies; namely, the technical methodology exhibits the best performance when using only ML predictions, with around 1978% cumulative returns with monthly rebalancing. In contrast, the fundamental methodology achieves its full potential when it is based primarily on LLM-derived semantic insights. The Entropy methodology is improved by a balanced combination of both semantic and ML signals, thus highlighting the potential of LLMs to improve predictive power by offering interpretative context for complex market interactions. These findings highlight the strategic importance of tailoring the semantic–algorithmic fusion to suit the nature of the predictive data and the investment horizon, with significant implications for portfolio management and future research in financial modeling.

## 1. Introduction

In modern parlance, the blending of traditional investment techniques with Artificial Intelligence (AI) has emerged as a central factor in financial forecasting. The capacity of AI to sift through and pool vast amounts of structured as well as unstructured data has enabled researchers and practitioners to construct increasingly sophisticated models for forecasting stock returns. Of the core fields of financial analysis (fundamental analysis, technical analysis, and entropy or complexity modeling) there is a growing realization that AI holds the key to bolstering predictive power by adding greater precision, flexibility, and context sensitivity [1,2,3]. Basic analysis underlies long-run investment strategies, involving evaluation of a firm’s underlying value by scanning financial statements, economic factors, industry conditions, and macroeconomic conditions. Metrics widely used in that regard include price-to-earnings (P/E) ratios, return on equity (ROE), debt-to-equity ratios, and discounted cash flow techniques. While such metrics provide important visibility into a firm’s operational feasibility and long-run prospects, conventional basic analysis has come under criticism on account of its reliance on history and its lack of quick responsiveness to changes in conditions in the marketplace [4]. The application of AI to basic analysis through machine learning and language processing enables real-time evaluation of earnings releases, managerial statements, and macroeconomic discourse, thus providing a more dynamic and forward-looking analysis framework [5,6]. At the same time, technical analysis inevitably remains a data-driven approach, focused on sentiment in the movement of price, volume, and market indications to predict short-run movements. Moving averages, Bollinger Bands, MACD, and Relative Strength Index (RSI) are tools that help traders identify changes in momentum, reversals, and support/resistance. While technical analysis excels in recognizing patterns of market dynamics, it has long been criticized for its limited explanatory powers and its inability to account for the broader market context [7]. More recent developments in artificial intelligence, notably deep learning methods, have greatly expanded the accuracy and automation of technical analysis by uncovering complex nonlinear relationships and enabling identification of patterns through copious amounts of time series data. The implications of these advances have led towards improved real-time decision-making powers and greater responsiveness within high-frequency trading environments.

Alternative methods for forecasting stock returns include fuzzy logic, which represents a powerful tool for treating the ambiguity and uncertainty of financial variables. Although not a substitute for binary logic, fuzzy logic enables reasoning for situations where variables cannot be clear-cut, either true or false, but are situated on a continuum of truth values. This property makes fuzzy systems especially effective for assessing investor sentiment, speculative economic predictions, and imperfect market indicators. The Mamdani and Sugeno models, as well as the hybrid neuro-fuzzy methodologies like ANFIS, have been widely applied in financial forecasting, leading to improved precision under conditions of noise and nonlinearity [8,9]. When coupled with artificial intelligence and entropy-based approaches, fuzzy logic uncovers deeper insight into market intricacies, turbulence, and behavioral dynamics, features that are typically ignored by rigid deterministic approaches. Ayyildiz and Iskenderoglu [10] found that artificial neural networks, logistic regression, and support vector machine algorithms could predict the directional movements of all countries’ stock indices with an accurate rate of over 70%. The researchers pointed out that the lack of economic indicators, the study time frame, and the number of technical tools used are limitations to their work.

Previous research shows that machine-learning models can forecast stock returns using fundamental, technical, and entropy-based features. To our knowledge, no study has yet embedded large language model (LLM) representations within each of these feature sets or measured the incremental contribution of the LLM layer to each strategy individually. This paper is the first to present a hybrid AI framework that combines the semantic context captured by transformer-based LLMs with conventional machine-learning pipelines for stock-return prediction.

The results are astounding: our technical approach, optimized in a monthly rebalanced context, achieved a spectacular cumulative return of 1978% over traditional benchmarks. More importantly, our entropy approach, using fuzzy entropy for capturing the structural intricacies of the market, achieved a strong cumulative return of 701% through balanced weighting with artificial intelligence and machine learning (w=0.70).

The findings of this research have several key implications. First, we demonstrate that semantic context inclusion significantly improves predictive quality in cases of ambiguity and complexity, as supported by primary narratives and entropy measures. Second, our findings confirm that the effectiveness of semantic artificial intelligence relies on methodological frameworks, and there is little extra value derived from extremely reactive, machine learning-optimized models, but considerable benefits are found with techniques that emphasize long-run goals and complexities. Third, we propose a scalable and modular approach that should help practitioners and researchers customize AI-augmented models for congruence with the strategic focus of their investment methodologies. Our findings provide strong empirical evidence for the prudent application of semantic-aware AI in financial modeling, supporting a shift towards contextually aware and multimodal investment approaches over established machine learning uses.

To ground our investigation, we focused on the NASDAQ-100 index, a data-rich, innovation-driven set of firms that offers both structured financial disclosures and abundant textual information for semantic analysis. The NASDAQ-100 presents an especially suitable environment for testing LLM-enhanced forecasting methods due to its concentration of well-documented, large-cap growth companies. We also chose the 2020–2025 time frame deliberately, as it captures a dynamic and uncertain period in recent market history, spanning the COVID-19 pandemic, inflationary surges, and monetary policy shifts. These conditions allow us to assess how hybrid semantic–algorithmic models perform not only in stable environments, but also under extreme and evolving market dynamics. From a practical standpoint, our models simulate stock selection within the index rather than replicating it, reflecting how active investors may apply AI-based tools in real-world decision-making.

## 2. Literature Review

The intersection of AI with fundamental and technical analysis has gained substantial scholarly attention, driven by its potential to enhance traditional forecasting frameworks. This section synthesizes key findings from recent literature, outlining how AI-based techniques augment conventional models across multiple investment approaches.

### 2.1. AI in Technical Analysis

The integration of AI into technical analysis has been widely studied, with consistent evidence supporting its predictive value. Souza et al. [11] showed that AI-enhanced moving average strategies can outperform market averages in BRICS countries. Neely et al. [12] demonstrated the viability of technical analysis using genetic programming in foreign exchange markets. While Dawson and Steeley [7] reported mixed results on the predictive power of visual chart patterns in the UK, their findings highlight the context-specific nature of technical trading rules. Machine learning has become instrumental in enhancing technical indicators. Shi and Zhao [3] used deep neural networks to model golden cross patterns, achieving superior predictive accuracy over a 10-year simulation. Yoo et al. [13] surveyed ML techniques that integrate event-driven variables, showing that qualitative information can enhance the technical framework. In line with this literature, our study demonstrates that ML-driven technical strategies excel under short-term rebalancing conditions, with the highest cumulative return (1978%) observed in our monthly-optimized configuration. However, unlike previous studies, we also test the added impact of semantic enrichment and find minimal marginal benefit in the purely technical context, reaffirming that traditional technical models may already capture most short-term signals efficiently. Abraham et al. [14] proposed utilizing a genetic algorithm combined with a Random Forest classifier to forecast daily stock trends. Their focus on predicting both uptrends and downtrends suggests that different algorithms might have varied efficiency depending on prevailing market conditions. Kabbani and Usta [15] address stock trend prediction by integrating news sentiment analysis with technical indicators. Their methodology reinforces the notion that understanding market context (uptrend vs. downtrend) can significantly improve predictive capabilities. Like these researchers, we reexamined the strategies during the stocks’ downfall period. This stress-test analysis reinforces the internal robustness of the scoring framework and confirms that the performance, reported as aggregate results, does not reflect bullish trends. Instead, it includes genuine exposure to high-risk, high-uncertainty periods where market structure and investor behavior were highly disrupted, a critical benchmark for evaluating practical performance in real-world investment settings.

### 2.2. AI in Fundamental Analysis

Incorporating AI into fundamental analysis has yielded promising improvements in stock return prediction. Agusta et al. [5] showed that recent financial indicators significantly improved the performance of MLP-based models. Zhou and Faff [6] argued that combining cross-sectional and time-series fundamental data enhances predictive accuracy, while Mokhtari [4] highlighted AI’s ability to transform traditional approaches through adaptive learning from macroeconomic indicators. Our findings align with these studies but add a unique layer: the semantic AI augmentation of fundamental models was particularly impactful in our framework. AI-derived contextual interpretation of earnings calls, regulatory filings, and macroeconomic commentary enhanced the richness and timeliness of fundamental insights, validating the critical role of unstructured data in long-term valuation.

### 2.3. Sentiment Analysis and NLP Applications

Sentiment analysis has seen rapid advancement through NLP models, enabling real-time monitoring of market mood. Liu et al. [16] developed prompt-based NLP systems for low-data scenarios, while Yan and Wang [17] proposed Attention-BiLSTM models to improve the granularity of sentiment prediction. Sufi and Khalil [18] introduced high-accuracy multilingual sentiment monitors, and Yang [19] demonstrated the profitability of analyst report sentiment tracking through hedged portfolios. Unlike prior work that isolates sentiment signals, our model integrates semantic indicators directly into scoring frameworks across all three investment strategies. This approach is particularly effective in entropy-based models, where semantic inputs help contextualize complexity indicators—resulting in a well-balanced AI-ML hybrid that achieved a 701% cumulative return, demonstrating enhanced interpretability and resilience.

### 2.4. Fuzzy and Entropy-Based Approaches

Fuzzy logic and entropy models have gained prominence for their ability to capture the ambiguous and nonlinear behavior of financial markets. Hurriyati et al. [8] and Alizadeh et al. [9] highlighted the strength of ANFIS in managing market uncertainty. Wu and Zhang [20] introduced fuzzy entropy as a robust measure for comparing financial time series, while Zhou et al. [21] demonstrated its utility in optimizing portfolios under uncertainty. Building on this, our entropy strategy incorporates fuzzy entropy within a semantic-aware AI model, offering a novel synthesis that is not present in previous work. The results reveal that semantic context helps disambiguate complex structural signals, allowing for more stable performance in high-entropy environments.

### 2.5. Comparative and Hybrid Modeling

Finally, the growing demand for hybrid modeling is supported by studies such as that by Ramesh et al. [22], who explored trust in AI-based robo-advisors, and Singh [23], who showed AI’s positive impact on financial performance. Zhao [24] confirmed the effectiveness of adapting AI-based strategies to sentiment conditions, supporting our finding that the optimal AI–ML blend depends on the investment horizon and strategy type. Our contribution extends this line of inquiry by empirically evaluating blended scoring models across three strategic axes, offering quantitative evidence of when and where semantic AI creates the most value. The result is a nuanced, adaptable framework that aligns model complexity with market complexity, setting a foundation for the next generation of hybrid predictive systems in finance.

## 3. Methodology—LLM-Created Scoring Framework

This section outlines a series of predictive scoring systems that were designed and developed entirely by a large language model (LLM), ChatGPT-4o, to simulate how such a model might independently evaluate stock quality and return potential using structured financial data. The LLM did not rely on human-defined investment heuristics or pre-existing financial models; rather, it selected the features, preprocessing steps, learning algorithms, and score transformations based on its autonomous interpretation of the problem. These scoring systems represent a novel form of collaborative machine–human research, in which the LLM serves not only as a computational assistant, but also as an analytical architect.

The methodology is composed of three distinct strategies, each reflecting a different domain of financial reasoning. The first, LLM-Constructed Fundamental Strategy, focuses on extracting latent performance signals from company income statements. The second, LLM-Constructed Technical Strategy, identifies predictive patterns in price behavior and short-term return dynamics. The third, LLM-Constructed Entropy Strategy, approximates fuzzy entropy using volatility-based stability proxies. In each case, the scoring model is trained on forward-looking returns and updated monthly and quarterly across a rolling 5-year prediction window, from 1 January 2020 until 1 January 2025. Final scores are normalized to the [0, 1] interval and serve as the decision layer for the portfolio selection process.

### 3.1. LLM-Constructed Fundamental Strategy—Learning from Financial Performance Signals

The fundamental strategy is based on the premise that a firm’s financial quality, as captured in its income statement, contains predictive information about future stock returns. Rather than relying on static, rule-based screens, we construct a supervised learning model to discover the implicit mapping between financial fundamentals and future performance. This allows us to capture complex, nonlinear interactions between accounting variables and market behavior.

We utilize quarterly income statement data for all NASDAQ-100 companies, obtained from Alpha Vantage, covering the period from 2019 through 2025. From these statements, we extract five features that capture profitability and operational efficiency: total revenue, operating income, net income, operating margin (operating income over total revenue), and net margin (net income over total revenue). These variables are chosen for their interpretability and established relevance in financial analysis.

Each data point is labeled with the corresponding 1-month and 1-quarter forward returns, based on adjusted close prices, forming two prediction targets for monthly and quarterly models, respectively. The target return is always aligned with the most recent financial report available at the time of prediction, ensuring that our models mimic the information set accessible to real-world investors and avoid lookahead bias.

We employ a multi-layer feedforward neural network to learn the relationship between the five input features and forward returns. The network architecture consists of two hidden layers with 32 and 16 neurons, respectively, using ReLU activation and trained using the Adam optimizer with a maximum of 500 iterations. Data are normalized using a StandardScaler to improve convergence and comparability across features. The model is trained on a pooled panel of all tickers, allowing it to generalize across sectors while still capturing firm-level signals.

Model predictions are transformed into LLM-style scores by clipping the output to the [0, 1] interval and applying percentile normalization where necessary. These scores represent the neural network’s assessment of the relatively expected return of each stock, given its current financial condition. Separate models are trained for the monthly and quarterly horizons to reflect potentially different relationships between fundamentals and short-term versus medium-term returns.

### 3.2. LLM-Constructed Technical Strategy—Learning from Price Behavior and Return Patterns

The technical strategy focuses on extracting predictive signals from the time-series behavior of stock prices. Unlike the fundamental strategy, which relies on accounting data, this approach seeks to learn patterns in recent returns, momentum, moving averages, and volatility that may indicate directional persistence or mean reversion.

For each stock, we construct a set of features using daily adjusted close prices. These features include lagged returns over 1-, 3-, and 6-month windows; moving averages over 3 and 6 months; and realized return volatility over the same horizons. The features are computed by resampling the daily data at the beginning of each month (for the monthly model) or each quarter (for the quarterly model). To avoid instability due to missing values or illiquid stocks, forward returns over the next month and next quarter are calculated using the same price resampling method and used as labels for supervised learning. The feature set is standardized, and a Ridge regression model is trained to predict forward returns. Ridge regression is selected for its balance between interpretability and performance and to mitigate overfitting in the presence of potentially collinear features.

Once trained, the model produces a score for each stock and date, which is normalized using a robust percentile-based method. Final technical scores fall within [0, 1] and represent the model’s learned estimate of positive return momentum and price strength. These scores are updated monthly or quarterly, depending on the portfolio rebalancing horizon, and reflect evolving return structures across time.

### 3.3. LLM-Constructed Entropy Strategy—Approximating Predictability via Volatility-Based Models

The third strategy draws inspiration from the concept of fuzzy entropy, which quantifies the degree of uncertainty or disorder in a system. In financial markets, entropy-based models aim to identify stocks whose return dynamics exhibit high stability and low randomness, characteristics that are thought to be associated with more predictable and reliable performance.

To approximate fuzzy entropy in a data-driven way, we use rolling return volatility as a proxy for signal stability. Specifically, we compute the standard deviation of daily returns over 21-day and 63-day windows, which represent recent short- and medium-term return behavior. These features are updated monthly or quarterly to align with the rebalancing periods of our portfolio strategies.

The relationship between return stability and future returns is modeled using Ridge regression. As with the technical strategy, this choice provides interpretability and robustness against noisy or sparse data. The regression is trained using forward returns as the prediction target, and regularization strength is chosen via internal validation. By learning how recent return volatility relates to future performance, the model produces a score that captures the structure embedded in low-entropy price movements.

Predicted return scores are normalized to the [0, 1] range, with higher scores corresponding to lower volatility and more predictable price behavior. This process operationalizes the entropy concept without requiring subjective membership functions or handcrafted thresholds. The resulting entropy-style score serves as a quantitative measure of signal clarity, suitable for integration into model-driven portfolio selection.

### 3.4. Construction of Algorithmic Scores and Hybrid Portfolio Optimization

This section outlines the construction of three machine learning-based predictive scoring systems for monthly and quarterly stock selection: one based on financial fundamentals, one based on technical indicators derived from price dynamics, and one based on entropy-driven complexity analysis. Each strategy aims to extract a distinct informational signal from historical or structural properties of firms, allowing us to capture orthogonal alpha streams. These algorithmic scores are later blended with large language model (LLM)-based scores, described earlier, to create hybrid stock ranking systems. The weights of these combinations are systematically optimized using historical return data.

The fundamental strategy draws on the well-established relationship between corporate performance and stock returns. For each company in the NASDAQ-100, we collected annual and quarterly financial statement data for a period of 5 years. The features extracted from each report included total revenue, gross profit, operating income, EBITDA, EBIT, net income, R&D expenditures, SG&A expenses, and interest-related metrics. From these, we derived financial ratios such as profit margins, operating leverage, R&D intensity, and return on equity. To mitigate the temporal sparsity of accounting releases, we added a feature encoding the number of days since the most recent report. This helped the models adjust confidence levels based on the freshness of the information.

A particularly innovative component of this strategy was the use of predictive modeling to estimate forthcoming report values before they were publicly released. We trained XGBoost regression models to predict each key financial metric using time series lags and trend-based features. XGBoost, an optimized implementation of gradient boosting, was selected for its ability to handle missing data, high-dimensional inputs, and complex nonlinear interactions without requiring heavy preprocessing. The model was trained on rolling windows, and predictions were generated recursively for each fiscal period to simulate a real-world forecasting scenario. These forward-looking fundamentals were then included in the feature set for a second layer of prediction, which estimated future stock returns over the next 21 or 63 trading days.

Once the enhanced feature set was finalized, combining both actual and predicted fundamentals, we proceeded to train a series of machine learning models to estimate forward stock returns. These return labels were computed over two investment horizons: 21 trading days (approximately 1 month) and 63 trading days (1 quarter). To maintain consistency with real-world investment constraints, only data available up to the return prediction start date were used, and the forward returns were clipped to control for extreme outliers. This ensured that the learning process focused on realistic return behavior and mitigated distortions from one-off price shocks.

The core return prediction step involved training four distinct regression models: Ridge regression, XGBoost, Random Forest, and a multi-layer perceptron (MLP). Each model was chosen to capture a different structural view of the input–output relationship. Ridge regression provided a regularized linear baseline that handled correlated features well, which was particularly important given the use of overlapping financial ratios and forward-looking forecasts. XGBoost and Random Forest offered complementary tree-based approaches, the former optimized for complex, nonlinear interactions, and the latter for robust, variance-reduced predictions across noisy or heterogeneous firms. The MLP neural network introduced an additional layer of flexibility by modeling subtle nonlinear trends across the entire input space, especially when patterns were smooth but not easily captured by trees or linear models.

To ensure that each model was both stable and effective in its predictions, we applied tailored optimization procedures suited to the nature of each algorithm. Rather than performing exhaustive hyperparameter searches, we adopted a pragmatic approach that balances performance, generalization, and reproducibility.

For Ridge regression, we used the RidgeCV implementation, which automatically selects the regularization strength (alpha) by performing internal cross-validation. The candidate values tested were 0.1, 1.0, and 10.0, a range chosen to explore both light and moderate regularization. This allowed the model to adaptively control the influence of correlated features without discarding informative predictors, which is especially important when working with financial ratios and their forward-estimated counterparts that often move in tandem.

In the XGBoost model, we configured a conservative setup with 100 boosting rounds, a maximum tree depth of 3, and a learning rate of 0.1. These parameters were chosen to prevent overfitting while preserving the algorithm’s ability to learn nonlinear and interaction effects. Since the feature set included both raw and derived metrics, this depth provided sufficient model complexity without compromising stability across different firms or time periods. The objective function used was squared error, aligning with our continuous return prediction targets.

For the Random Forest, we trained 100 decision trees with a maximum depth of 6. This relatively shallow depth was selected to avoid overfitting to firm-specific noise and to encourage general patterns to dominate across trees. Random subsampling and feature bagging were used by default, further reducing the risk of model variance and making the ensemble particularly well-suited for absorbing the irregularities present in financial data across firms and time.

The multi-layer perceptron (MLP) was configured with two hidden layers of 32 and 16 neurons, respectively, using ReLU activation functions to introduce nonlinearity. The model was trained using the Adam optimizer, with a maximum of 500 iterations to allow for convergence without excessive training time. Preprocessing was handled using a “Standard-Scaler” within a pipeline to ensure that all input variables were appropriately scaled, a critical step in neural network training. The model also used a fixed random seed to maintain reproducibility and consistent convergence behavior.

Together, these carefully selected configurations allowed each model to contribute unique insights while maintaining overall robustness and compatibility within the ensemble structure.

To ensure stability and comparability across models, all inputs were standardized using “Standard-Scaler”, and model outputs were transformed using a percentile-based normalization scheme. For each model, raw return predictions were scaled into the [0, 1] interval after clipping values at the 1st and 99th percentiles. This allowed each model to contribute meaningfully to the final score without being skewed by scale or outliers. The normalized predictions were then blended using fixed weights: 30% each for Ridge and XGBoost, and 20% each for the MLP and Random Forest. These weights were selected based on internal validation and practical considerations of model diversity and risk control.

The result of this ensemble procedure is a single, unified score, the monthly (or quarterly) fundamental score, that reflects the synthesized view of multiple learning algorithms operating over a rich, forward-augmented fundamental dataset. This approach allows the strategy to dynamically respond to changes in firm fundamentals, both actual and anticipated, while leveraging the interpretability of structured financial features and the predictive power of diverse machine learning architectures.

The technical strategy employed a set of momentum and volatility indicators calculated from historical daily adjusted closing prices. Standard technical indicators such as Relative Strength Index (RSI), Moving Average Convergence Divergence (MACD), simple and exponential moving averages (SMAs, EMAs), and rolling standard deviation were used. These features were calculated over multiple windows (7, 14, and 21 days) to capture short-term and medium-term price dynamics. Rather than relying on a single predictive model, we adopted an ensemble approach comprising XGBoost, LightGBM, CatBoost, and an LSTM (Long Short-Term Memory) neural network.

Each model in the ensemble served a distinct purpose. The gradient boosting models (XGBoost, LightGBM, CatBoost) are tree-based learners that perform well with tabular data and are capable of modeling nonlinear interactions among features. LightGBM was particularly effective in high-dimensional settings due to its leaf-wise growth strategy and histogram-based feature binning. CatBoost was selected for its categorical feature handling and robustness against overfitting on small training sets. The LSTM, a type of recurrent neural network (RNN), was incorporated to learn from sequential dependencies in the input features. LSTMs are designed to preserve long-range memory through gated recurrent units, making them well-suited for modeling temporal dynamics and recurrent trends in financial time series. In our implementation, we trained LSTM models on 30-day rolling sequences of technical indicators and used them to predict 21- or 63-day forward returns. The outputs of all models were averaged to produce the final technical score.

The entropy strategy leverages fuzzy entropy, a nonlinear time series analysis tool that quantifies the degree of complexity or uncertainty in a system. Traditional entropy metrics such as Shannon entropy or approximate entropy require fixed-length sequences and suffer from sensitivity to noise or small fluctuations. Fuzzy entropy addresses these limitations by using a fuzzy membership function to determine the similarity between sequences. It replaces the binary similarity used in approximate entropy with a continuous-valued function, which reduces discontinuities and enhances robustness in noisy environments such as financial markets.

Mathematically, fuzzy entropy is calculated by first forming vectors $x_{i}\in R^{m}$ from a time series $X=\{x_{1},x_{2},...,x_{N}\}$ using embedding dimension m. For each vector $x_{i}$, the similarity to all other vectors $x_{j}$ is measured using a fuzzy function

(1) $$D_{ij}=exp\left(-\frac{\left(∥x_{i}-x_{j}{∥}^{n}\right)}{r}\right)$$

where r is a tolerance threshold and n is a fuzzification parameter (typically set to 2). The average similarity is computed for all pairs, and the entropy is defined as the natural logarithm of the ratio of average similarities for vectors of length m versus m+1. The final entropy value reflects how predictable local patterns in the data remain when extended by one period step.

In our implementation, we applied fuzzy entropy to rolling windows of 30 trading days for each stock, generating a dynamic sequence of entropy values over time. These values were used directly as features or in conjunction with other statistical summaries (mean, variance, trend) to form a predictive vector. As with the technical strategy, we fed this vector into the same ensemble of models (XGBoost, LightGBM, CatBoost, LSTM), each of which predicted expected returns over the next investment horizon. The ensemble output formed the entropy-based score for each stock.

After obtaining predictive scores from the three algorithmic strategies, we combined them with the LLM-generated scores using a linear weighted average. For each strategy, the final combined score was computed as

(2) $$CombinedScore_{i,t}=w\cdot MLScore_{i,t}+\left(1-w\right)\cdot LLMScore_{i,t}$$

where w is a blending weight assigned to the ML score, ranging from 0 to 1 in increments of 0.05. For each candidate weight, we conducted a portfolio simulation across the full backtest period. On each rebalancing date (monthly or quarterly), we selected the top 10 stocks with the highest combined score, formed an equal-weighted portfolio, and held it for the corresponding horizon (21 days or 63 days).

The returns of these portfolios were recorded and used to compute performance statistics, including average return per holding period, standard deviation (volatility), Sharpe ratio (assuming a zero risk-free rate), and cumulative return. The blending weight that yielded the highest cumulative return was identified as the optimal value for each strategy and frequency. For each optimal configuration, we retained the full return time series and the set of selected stocks per rebalancing period.

This comprehensive approach allowed us not only to compare the standalone performance of each algorithmic strategy, but also to quantify the added value of integrating LLM-derived semantic signals. It further enabled a systematic, data-driven selection of combination weights that maximize portfolio performance, offering a robust and extensible framework for hybrid investment strategies.

It is important to note that, although our analysis is based on historical data from 2020 to 2025, the modeling process was designed to reflect real-time investment conditions. At each rebalancing point, models were retrained using only data available up to that date, and forward returns were aligned to reflect future performance. This rolling-window approach ensures that each portfolio decision was made using a realistic information set, avoiding lookahead bias and simulating out-of-sample behavior. While this validation framework improves robustness within the tested period, we recognize that full generalization to future or external markets would require further testing, a direction we highlight in the conclusion as a valuable opportunity for future research.

## 4. Results

This chapter provides a comprehensive comparison of the performance of three independent forecast methods––fundamental, technical, and entropy––applied to NASDAQ-100 stocks for the 5-year forecast horizon from 2020 through 2025. All three methods incorporate algorithmic scores generated from machine learning (ML) models, together with semantic scores based on a large language model (LLM). The weighting parameter (w) in our composite scoring system signifies the proportion assigned to forecasts based on ML, while the remaining proportion, 1−w, quantifies the degree of reliance placed on the interpretations derived semantically from the LLM.

The results presented in Table 1 show substantial differences in the optimum machine learning weight (w) between methodologies and rebalancing frequencies, that is, between monthly and quarterly. The technical approach performed best under monthly rebalancing conditions when solely based on machine learning predictions (w=1), with a cumulative return of around 1978% over the course of the study. The fundamental approach, by way of comparison, performed best on a monthly basis, with a machine learning weight of only 0.15, highlighting the substantial benefits of employing scores based largely on semantic large language models (LLMs). The entropy method, which evaluates the fundamental attributes of stock price movements, performed best with an equal weighting approach (w=0.70), thus highlighting its improved predictive power through a hybrid scoring framework that combines the sophisticated analyses of both machine learning and LLM approaches.

Examination of the quarterly rebalancing horizon highlights the crucial importance of successfully blending algorithmic and semantic scores. Compared with the quarterly technical approach, maximum performance was achieved using a machine learning weight of 0.45, yielding a total return of around 573%, coupled with a stellar Sharpe ratio of 1.297, the highest reported by any approach and time horizon. This report suggests that the addition of contextual intelligence from big language models significantly improves sustainability and reduces risk over long periods of investment without sacrificing significantly in terms of return. By comparison, the entropy approach had the most impressive performance in the quarter, with an equivalent intermediate machine learning weight of 0.40, indicating that the blending of semantic analysis and entropic metrics uniformly improves predictive effectiveness over long time periods. It should be noted that the fundamental quarterly approach performed optimally, with the most reliance on scores from big language models (w=0), thus proving that semantic trackers associated with fundamental financial metrics occasionally perform better than structured algorithmic designs over extended horizons.

**Figure 1:**
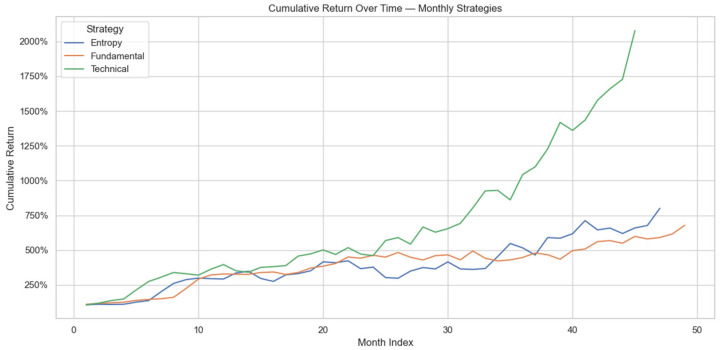
Cumulative return over time for top monthly strategies; fundamental, technical, entropy.

The cumulative return trajectories illustrated in Figure 1 offer deeper insight into the month-by-month evolution of the monthly strategies. Early on, all strategies exhibited relatively similar performance; however, after approximately 2.5 years (around month 30), the monthly technical strategy diverged significantly upward, vastly outperforming its fundamental and entropy counterparts. This pronounced divergence emphasizes the technical strategy’s ability driven purely by ML to effectively exploit momentum, trend-following, and short-term market inefficiencies. In contrast, the more balanced entropy strategy and heavily semantic-driven fundamental strategy demonstrated steadier but less explosive growth, suggesting that the value of semantic context and complexity measures lies more in risk moderation and stable incremental gains rather than aggressive return generation.

**Figure 2:**
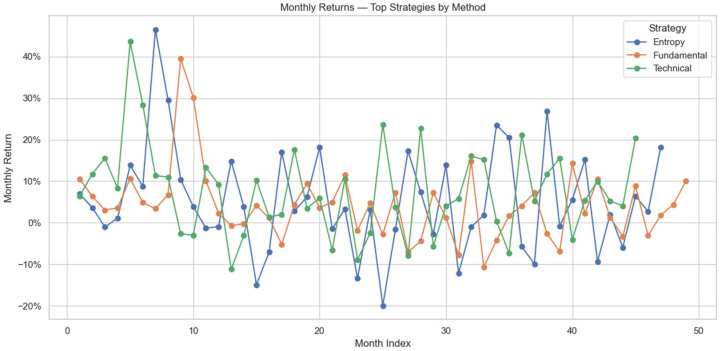
Monthly return volatility of top monthly strategies by method.

The month-to-month return variation presented in Figure 2 provides further insight. The technical approach clearly displays higher volatility, reflecting the higher, more aggressive nature of pure machine learning methods that take advantage of rapid market movements. The fundamental approach, on the other hand, reflects less monthly volatility, reflecting its more conservative, fundamentally based approach augmented with the semantic understanding provided by the large language model. The entropy approach falls in between, reflecting the optimal balance of machine learning parameters that determine its equilibrium between return and volatility, reflecting effective blending of market structure analysis with semantic context.

**Figure 3:**
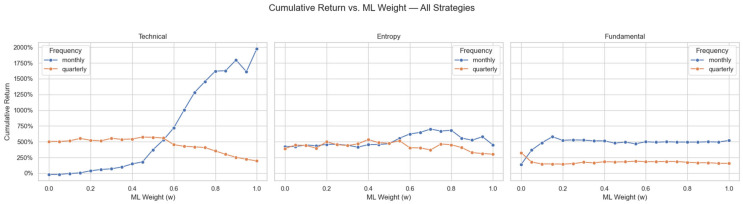
Cumulative return as a function of ML score weight (w) for each strategy.

Analysis of cumulative returns over various machine learning weights (w), as plotted in Figure 3, reveals interesting results regarding optimal blending approaches for each methodology. The technical method returns rise steadily in tandem with increasing ML weights, reaching 1 for monthly and 0.45 for quarterly measurements, thus clearly showing its affinity with quantitative, price-based modeling methodologies. The near-total dependence on ML for monthly technical suggests minimal incremental benefit from semantic analysis in the context of short-horizon technical trading setups. In sharp contrast, the entropy method manifested a clear concave relation, indicating diminishing returns at both extremes. Optimal performance was seen in a finely balanced mix of semantic inputs, revealing that large language model inputs materially enhance predictive power when analyzing intricate market dynamics. The fundamental strategy shows a very different profile, with returns rising sharply to their peak at lower ML weights, reflecting the profound influence that semantic interpretations can exert over conventional financial fundamentals. Indeed, in the case of fundamental analysis, the large language model’s ability to contextually read earnings reports, management guidance, macroeconomic sentiments, and industry trends provides significant incremental predictive power over purely numerically driven algorithms.

**Figure 4:**
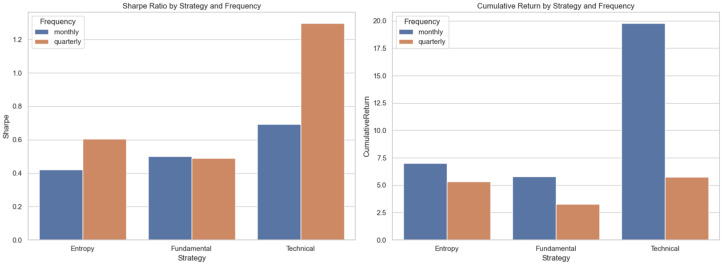
Sharpe ratio and cumulative return comparison by strategy and frequency, monthly vs. quarterly.

Figure 4, showing an overview of Sharpe ratios alongside cumulative returns for both the monthly and quarterly strategies, further substantiates these findings. It identifies the key risk–return trade-offs involved in the choice of strategies and machine learning weighting. Although the monthly technical strategy delivered impressive cumulative returns, its Sharpe ratio was significantly weaker than that of the quarterly version, reflecting an elevated relative risk. Conversely, the quarterly technical strategy, by virtue of its integrated nature, maximized risk-adjusted returns, thus supporting the fact that semantic insights, when coupled with algorithmic models, not only render predictive precision but also successfully reduce investment risk over extended holding periods. Furthermore, both the entropy and fundamental strategies registered improvements in their risk-adjusted metrics when semantic analysis made a major contribution, substantiating the fact that the inclusion of qualitative LLM insights tends towards greater investment stability and efficiency.

In summary, extensive empirical analysis demonstrates that strategic integration of ML and LLM insights can significantly enhance portfolio management across varying investment horizons. Pure ML methods excel where price momentum and short-term quantitative signals dominate, whereas semantic LLM insights profoundly improve outcomes in areas where complex context and interpretation of unstructured data such as fundamental metrics and entropy measures are critical. These results reinforce the idea that the optimal balance between structured and semantic information depends strongly on the type of data and the temporal investment horizon considered, thus providing valuable guidance for investors and portfolio managers seeking to leverage modern predictive methodologies to maximize returns and optimize risk profiles.

### 4.1. Performance Under Market Stress: COVID-19 Crash Analysis

To evaluate model robustness under extreme market conditions, we isolate the early 2020 COVID-19 crash, a period of historic volatility and rapid market correction. Between February and May 2020, the NASDAQ-100 and broader indices experienced one of the fastest and steepest drawdowns in modern financial history, triggered by uncertainty around the pandemic and global economic shutdowns.

**Figure 5:**
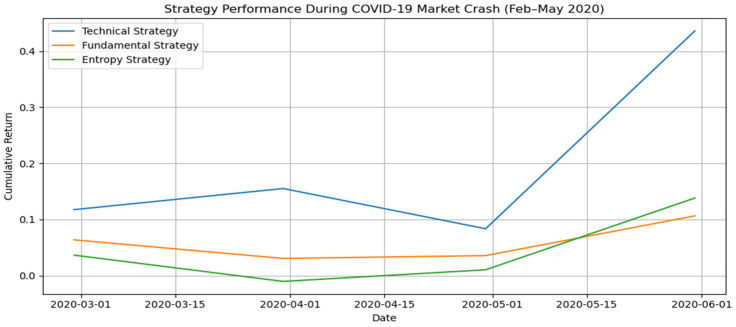
Cumulative return over time for top monthly strategies, fundamental, technical, entropy, at the beginning of the COVID-19 pandemic.

Figure 5 presents the cumulative return performance of the three strategy models, fundamental, technical, and entropy, during the February to May 2020 window. The data clearly illustrate that all models encountered a turbulent environment, with sharp changes in momentum and volatility. Importantly, none of the strategies collapsed or ceased to function during this period, and all three displayed clear differentiation in behavior and recovery dynamics.

The technical strategy initially outperformed during the early stages of the crash and was quick to rebound by May, suggesting its responsiveness to short-term price reversals. The fundamental and entropy strategies both showed more muted movements, reflecting a more conservative profile and perhaps some lag in adjusting to the new macroeconomic signals. Notably, while the fundamental model exhibited a modest decline through March and April, it began to recover as forward-looking indicators stabilized, demonstrating the framework’s ability to respond to changing market signals even under stress.

This stress-test analysis reinforces the internal robustness of the scoring framework and confirms that the performance reported in aggregate results does not merely reflect bullish trends. Instead, it includes genuine exposure to high-risk, high-uncertainty periods where market structure and investor behavior were highly disrupted, a critical benchmark for evaluating practical performance in real-world investment settings.

## 5. Concluding Remarks

The analysis demonstrates the tremendous potential of integrating ideas of large language models with ordinary machine learning techniques in forecasting investments. By altering the ML weights of blended scoring techniques based on the application of fundamental, technical analysis, and entropy-based techniques, we have provided strong evidence of when and where meaning contributes towards conventional numerical signals.

The technical ML approach performed best on a monthly rebalancing schedule. It achieved returns around 1978% in total. The entropy approach was created to quantify the degree of market intricateness through fuzzy entropy calculations. It proved its worth through consistent and decent returns, totaling around 701%, and equal weighting for ML and LLM (w=0.70). The entropy approach proved with ease that it could manage market intricateness. It demonstrated the significance of the concept of meaning in structural intricateness measurement, adding context that other simple approaches do not.

We see that the additional value of semantic context for use in LLMs varies greatly according to prediction method and horizon. Specifically, ML methods will work better for the short horizon, when technical nuances and rapid shifts in data matter most. Conversely, the unambiguous benefit of semantic understanding demonstrates how semantic models can extract beneficial knowledge from verbose financial statements, major company announcements, and broad economic narratives.

The balanced optimization observed in entropy strategies suggests that LLMs provide essential interpretive layers to market complexity measures, effectively bridging structural market indicators with sentiment and narrative information.

From a practical investment perspective, our results have clear implications. Portfolio managers focusing on short-term, momentum-driven strategies may continue relying on robust ML-based models, reserving semantic insights for tactical enhancements. In contrast, those emphasizing fundamental analysis should strongly consider allocating greater predictive responsibility to semantic-driven methods. For strategies grounded in market complexity and behavioral insights, hybrid models equally blending ML and LLM inputs appear ideal, offering an effective balance of precision and context.

Overall, this research illustrates the potential of semantic-aware machine learning in finance, advising practitioners and scholars to embrace hybrid predictive approaches that leverage the full analytical power of structured algorithmic methods alongside the interpretive capabilities of modern LLMs.

While the results are promising, it is important to acknowledge certain limitations. First, the study focuses on NASDAQ-100 stocks, which may limit generalizability to other indices or international markets. However, the methodology itself is adaptable to broader universes. Second, although our portfolio simulations are based on historical data, the use of rolling-window forecasting ensures that all predictions are made using information available at the time, thereby mimicking realistic, out-of-sample performance and avoiding lookahead bias. In addition to using a rolling-window forecasting framework, we further validated the robustness of our approach by isolating the COVID-19 crash period. This analysis, presented in Section 4.1, confirmed that the models remained functional and responsive even during one of the most volatile and uncertain market environments in recent history. The differentiated behavior across strategies during this period supports the view that the framework can adapt to dynamic market conditions, not just trending environments.

Compared to earlier studies like Ayyildiz and Iskenderoglu (2024) [10], which focus on classification accuracy across indices, our work takes a more applied perspective by linking predictive outputs to actual portfolio construction. This enables us to assess not only the statistical strength of our models, but also their investment utility over time.

Future research could extend this hybrid modeling framework to different markets, asset classes, or shorter holding periods. Practitioners may also explore how the integration of macroeconomic variables or real-time news sentiment impacts predictive performance. Ultimately, our study demonstrates how LLM-augmented models can bridge the gap between advanced AI capabilities and practical financial decision-making.

## Figures and Tables

**Table 1:** Optimal ML weights, risk-return metrics, and cumulative returns for all strategy-frequency combinations (2020–2025).

Strategy	Frequency	ML Weight	Sharpe	Average Return	Volatility	Cumulative Return
Technical	monthly	1	0.6934	7.50%	0.1082	1977.71%
Entropy	monthly	0.7	0.4207	5.23%	0.1244	700.52%
Fundamental	monthly	0.15	0.5001	4.32%	0.0863	578.40%
Technical	quarterly	0.45	1.2967	24.99%	0.1927	573.37%
Entropy	quarterly	0.4	0.6048	20.25%	0.3348	534.36%
Fundamental	quarterly	0	0.4899	14.71%	0.3002	326.12%

## Data Availability

The raw data supporting the conclusions of this article will be made available by the authors on request.
